# Neurovascular coupling during auditory stimulation: event-related potentials and fNIRS hemodynamic

**DOI:** 10.1007/s00429-023-02698-9

**Published:** 2023-09-02

**Authors:** Vanesa Muñoz, Manuel Muñoz-Caracuel, Brenda Y. Angulo-Ruiz, Carlos M. Gómez

**Affiliations:** 1 Human Psychobiology Laboratory, Experimental Psychology Department, University of Sevilla, Seville, Spain; 2 Hospital Universitario Virgen del Rocio, Seville, Spain

**Keywords:** Auditory stimulation, Neurovascular coupling, IDAP, fNIRS, Auditory cortex, ERPs

## Abstract

Intensity-dependent amplitude changes (IDAP) have been extensively studied using event-related potentials (ERPs) and have been linked to several psychiatric disorders. This study aims to explore the application of functional near-infrared spectroscopy (fNIRS) in IDAP paradigms, which related to ERPs could indicate the existence of neurovascular coupling. Thirty-three and thirty-one subjects participated in two experiments, respectively. The first experiment consisted of the presentation of three-tone intensities (77.9 dB, 84.5 dB, and 89.5 dB) lasting 500 ms, each type randomly presented 54 times, while the second experiment consisted of the presentation of five-tone intensities (70.9 dB, 77.9 dB, 84.5 dB, 89.5 dB, and 94.5 dB) in trains of 8 tones lasting 70 ms each tone, the trains were presented 20 times. EEG was used to measure ERP components: N1, P2, and N1–P2 peak-to-peak amplitude. fNIRS allowed the analysis of the hemodynamic activity in the auditory, visual, and prefrontal cortices. The results showed an increase in N1, P2, and N1–P2 peak-to-peak amplitude with auditory intensity. Similarly, oxyhemoglobin and deoxyhemoglobin concentrations showed amplitude increases and decreases, respectively, with auditory intensity in the auditory and prefrontal cortices. Spearman correlation analysis showed a relationship between the left auditory cortex with N1 amplitude, and the right dorsolateral cortex with P2 amplitude, specifically for deoxyhemoglobin concentrations. These findings suggest that there is a brain response to auditory intensity changes that can be obtained by EEG and fNIRS, supporting the neurovascular coupling process. Overall, this study enhances our understanding of fNIRS application in auditory paradigms and highlights its potential as a complementary technique to ERPs.

**Supplementary Information:**

The online version contains supplementary material available at 10.1007/s00429-023-02698-9.

## Introduction

In recent decades, the integration of techniques that study different physiological parameters has been proposed to gain an in-depth understanding of brain activity, considering the complexity of the human brain. An example of such integration is the combination of electroencephalography (EEG), which has high temporal resolution, with functional magnetic resonance imaging (fMRI) or functional near-infrared spectroscopy (fNIRS), which provides good spatial information (Mulert et al. 2005; Li et al. 2022). This new research paradigm promises to be a good approach to obtain more reliable and comprehensive results, which can be very useful in cognitive neuroscience (Herrmann et al. 2010). Considering the difficulty of analyzing the areas involved in the generation of ERPs due to volume conduction, fNIRS could be a good approach to add spatial information to the dynamical information provided by ERPs, since compared to fMRI is technically more adaptable for co-registration. In addition, the hemodynamic response depends on an indirect measure of blood oxygenation, leading to a delayed response to the stimulus. Thus, both techniques could be used together for a better and complementary understanding of cognitive processes (Mulert et al. 2005). However, co-recording also has some disadvantages. On the one hand, recording two signals at the same time can be more stressful for the subjects (Karch and Mulert 2010). On the other hand, in the design of the experiment, both the guidelines for the EEG and the fNIRS signals should be addressed. Since they have different response times, the intervals between stimuli and the properties of the stimuli or tasks should be adjusted to find the expected response.

The combination of electrical and hemodynamic brain response techniques is explained by a process called “neurovascular coupling”. This process involves brain electrical activity and increased cerebral blood flow in response to the presentation of a stimulus or series of stimuli (Villringer et al. 2010). Normal brain activity is continuously self-regulated by the cerebral arteries. They relax when blood pressure decreases and constricts during blood pressure increases, thus maintaining stable intracerebral pressure. In the presence of a stimulus or when a cognitive state is induced, coupled with cerebral electrical activation, there is a change in cerebral vascular activation that deviates from baseline. The change in normal activity is perceived as an increase in cerebral blood flow and is known as “functional hyperemia”. Neurovascular coupling refers to the mechanism by which neuroelectrical activity and functional hyperemia are coupled (Shmuel 2010). The neurovascular response is based on the assumption that neuronal activity requires large amounts of energy (glucose) and oxygen to generate ATP and maintain the mechanisms of neurotransmission and neuronal communication (Attwell et al. 2010). The widely accepted hypothesis is that activity-induced changes in neurons mediate neurovascular coupling. Neurotransmitters and *K* + are released as a result of synaptic activity, initiating and maintaining the neurovascular response (Longden et al. 2017). Studies in animals under anesthesia have proposed that neural activation leads to an increase in synaptic activity in the active region, resulting in the release of vasoactive agents from neurons and astrocytes. These agents cause blood vessel dilation (Schei et al. 2012).

fNIRS is a method of assessing brain activity by measuring the hemodynamic changes produced by brain activation. Brain activity is associated with various physiological processes that cause changes in the optical properties of brain tissue. Thus, fNIRS technology measures hemodynamic changes in the cerebral cortex through the “transparency” of tissue to near-infrared (NIR) light. NIR light with an appropriate wavelength can be absorbed by blood chromophores or scattered in tissues. The attenuation of light is primarily caused by the main chromophore in the brain: hemoglobin. In the brain, oxyhemoglobin (HbO) and deoxyhemoglobin (HbR) are usually the dominant absorbers, allowing optical methods to quantify the predominant hemodynamic variables (Ferrari and Quaresima 2012; Pinti et al. 2020).

Physiologically, this technique is based on the assumption that active neurons, through the process of neurovascular coupling, produce an increase in arterial blood flow in the surrounding vascular vicinity. This increase compensates for the consumption of glucose and oxygen caused by the postsynaptic activation and action potentials, which require high energy levels (Attwell et al. 2010). As a result, there is an oversupply of regional cerebral blood flow (CBF), leading to an increase in HbO and a decrease in HbR concentrations (Pinti et al. 2020). This phenomenon is known as the hemodynamic response function (HRF) and is typically characterized by a 2 s delay between the onset of the electrical neural response. These changes start with a steep rise, reaching a plateau approximately 6–10 s after the stimulus onset (van de Rijt et al. 2018). According to the neurovascular process, it has been proposed that following neural activation, there is a relaxation of the vascular musculature, resulting in increased blood flow to overcompensate for energy expenditure. This overcompensation or functional hyperemia is a fundamental phenomenon in normal brain function. It is defined as the dilation of arterioles and capillaries in a brain region in response to a local episode of high neural activity (Cinciute 2019).

fNIRS is an optimal tool for studying auditory stimulation paradigms due to its non-invasive and silent nature. In contrast, fMRI is often unsuitable for this purpose due to the constant noise produced by the instrument, which can interfere with the presentation of the stimulus (Gaab et al. 2007). Despite this limitation, most studies investigating the relationship between sound intensity or frequency and the activation of the auditory cortex have been conducted with fMRI. These studies have found an increase in the BOLD response in the auditory cortex with changes in the frequency and/or intensity of stimulation. Moreover, they have demonstrated a dependent change in amplitude with increases in intensity. These effects have generally been observed in the medial and lateral regions of the superior temporal gyrus (Hall et al 2001; Hart et al. 2003; Langers et al. 2007; Röhl and Uppenkamp 2012), as we all in the cochlear nucleus, inferior colliculus, medial geniculate body (Sigalovsky and Melcher 2006). Lateralization for processing of the level-dependent stimulation in the auditory cortex has also been suggested (Brechmann et al. 2002).

The few fNIRS studies on this topic show different results, supporting or contradicting the fMRI results. A review conducted by van de Rijt et al. (2018) suggests that fNIRS can be a useful tool for studying auditory paradigms. However, there are still many doubts about the modulation of the hemodynamic response to different sound characteristics, such as intensity, rates, sound complexity, frequency, duration, repetition, and attention. Some studies exclusively using fNIRS have shown significant changes in HbO and HbR, in response to shifts in frequency/intensity of auditory stimulation. Weiss et al. (2008) found a decrease in HbR with increasing stimulation rate, and Bauerfeind et al. (2016, 2018) observed cortical activation in temporal and frontal regions (medial temporal gyrus, orbital, triangular, and opercular parts) and deactivation in central and parietal regions (precentral gyrus and inferior parietal gyrus). The activation and deactivation patterns demonstrated dependent intensity amplitude changes. Similarly, Weder et al. (2018) found that higher intensity levels led to higher concentration changes, particularly in the superior temporal gyrus. They also observed differences in waveform patterns, with phasic responses near the supramarginal and caudal superior temporal gyrus, and tonic responses in channels over Broca's area. The same results were found by Weder et al. (2020), moreover, this study pointed out that the dependence on intensity changes may be better explained by the perception of the subjects (loudness) rather than intensity. This response being more pronounced in the superior temporal gyrus of the right hemisphere. Conversely, Muñoz-Caracuel et al. (2021) and Muñoz et al. (2022), found that the highest intensities (94.5 dB), could lead to a decrease in HbO concentrations, possibly due to systemic vasoconstriction. Furthermore, Chen et al. (2015) did not find an auditory intensity effect in the auditory cortex but found a modulation related to perceived intensity. This finding suggests, similar to Weder et al. (2020), that auditory cortical response studied with fNIRS would be more sensitive to perceived intensity than the physical property of sound intensity. In this regard, integrating EEG with fNIRS could be beneficial for studying sound attributes, as it would provide additional information about the processing of auditory stimulation with better temporal resolution.

EEG is a method for recording brain electrical activity through electrodes placed on the scalp. This recording captures the voltage change related to the sum of the synchronized electrical activity of neurons in the brain regions processing the stimuli (Rugg and Coles 1995). However, due to volume conduction, the final recorded signal reflects the sum of multiple activations propagating from nearby regions, resulting in poor spatial resolution (Michalopoulos et al. 2015). Event-related potentials (ERPs) are defined as changes in voltage within a specific temporal window and scalp location in response to a particular stimulus or motor response. Repeating the stimulation enables the detection of this response by averaging the recorded signals across trials, thereby increasing the signal-to-noise ratio (SNR). ERPs represent electric fields associated with populations of neurons, primarily large pyramidal neurons, which are synchronously active and aligned in a parallel orientation (open field). This alignment results in the electric fields of each neuron summing to produce a dipolar field with both positive and negative charges. It has been proposed that the waves recorded by ERPs reflect postsynaptic potentials instead of action potentials (Allison et al. 1986; Rugg and Coles 1995).

Auditory ERPs can be derived from EEG signals to assess changes in brain electrical activity in response to variations in sound wave amplitude or other parameters. The most commonly studied auditory ERPs are P1 (a positive deflection occurring between 40 and 60 ms after stimulus onset), N1 (a negative deflection occurring between 60 and 150 ms after stimulus onset), and P2 (a positive deflection occurring between 150 and 250 ms after stimulus onset) (Paiva et al. 2016). Näätänen and Picton (1987) proposed that the N1 component is associated with the detection and orientation to changes in the auditory stimulation and that it is composed of at least three underlying components with a topography near the primary auditory cortex, including Heschl’s gyrus, and the lateral part of the superior temporal gyrus (Näätänen and Picton 1987; Woods 1995). Likewise, some authors have proposed the recruitment of non-specific auditory areas such as the prefrontal and cingulate cortices (Giard et al. 1994; Gallinat et al. 2002; Zhang et al. 2011), this frontal activity seems to enable the brain to filter out irrelevant and repeated stimuli, and thus enhance the auditory sensitivity to the stimulation. The P2 auditory wave is linked to stimulus classification, attention targeting, perceptual learning, and inhibitory processes for irrelevant stimuli. The exact topography of the P2 component is still under debate, but it has been proposed that the neural substrates may include the mesencephalic reticular activating system, the planum temporale, and Broadmann’s Area 22 (Paiva et al. 2016).

Several studies have established a correlation between the intensity of the sound level and the amplitude of the N1 and P2 components, known as loudness dependence of auditory evoked potentials (LDAEP) or intensity-dependent amplitude changes (IDAP) (Hegerl et al. 1994; Dierks et al. 1999). Research suggests that the regulation of intensity dependence in the N1 and P2 components may be attributed to the auditory cortex, particularly layer IV, which exhibits a high concentration of serotonergic innervation (Hegerl and Juckel 1993; Hegerl et al. 1994, 2001). Thus, the intensity dependence of the auditory evoked components has been proposed as an indicator of central serotonergic activity. In the clinical population, the IDAP has been included in studies of depression and other psychiatric disorders such as bipolar disorder (Park and Lee 2013), and schizophrenia (Gudlowski et al. 2009) among others (Park et al. 2010). Suppression of intensity modulation in these disorders has been observed, potentially reflecting low central serotonergic neurotransmission (Hegerl and Juckel 2000). Studies evaluating the efficacy of medications, including selective serotonin reuptake inhibitors (SSRIs), in treating these disorders have shown promising results including the IDAP paradigm (Gallinat et al. 2000; Linka et al. 2005). In addition, the IDAP has been found to provide a differential prediction of response to different classes of antidepressants (Juckel et al. 2007).

Recent studies have used fNIRS and EEG in auditory stimulation paradigms, and in some cases have found correlations between both techniques, suggesting neurovascular coupling. For instance, Ehlis et al. (2009) found a positive correlation between the amount of sensory gating (gating quotient Q) and the strength of the hemodynamic response in the left prefrontal and temporal cortices during dual clicks Similarly, language studies have reported relationships between EEG measures and activation or lateralization of related brain areas (Horovitz and Gore 2004; Steinmetzger et al. 2022; Wallois et al. 2012). However, some studies have not consistently found such clear relationships, suggesting potential masking effects caused by cortical blood stealing (Steinmetzger et al. 2020), which is proposed could influence the fNIRS signal.

Studies investigating sound intensity have mainly used fMRI to correlate ERPs (N1, P2) and hemodynamic activity. These studies, alongside the known effect of amplitude change with intensity in N1, P2, and N1–P2, have found correlations between the intensity level and the number of active voxels in the primary auditory cortex. However, findings across studies are not consistent. While Mulert et al. (2005) found no relationship between BOLD signal amplitude and sound intensity level, Thaerig et al. (2008) reported an increase in both the number of voxels and BOLD signal amplitude at higher sound intensities. This response was not limited to the primary auditory cortex but was also observed in Heschl’s gyrus, the planum temporale, with lateralization to the right hemisphere. Similarly, Neuner et al. (2014) identified a broader range of activated areas with increasing intensity, involving the anterior cingulate cortex, opercular and orbitofrontal cortex. The increase in activation with intensity was most pronounced in Heschl's gyrus and the insular cortex. However, although both fNIRS and fMRI measure hemodynamic activity, fNIRS has spatial limitations compared to fMRI. Moreover, the commonly used continuous-wave fNIRS devices measure changes in concentration values rather than absolute values. Therefore, the findings should be interpreted with these limitations in mind. To our knowledge, Chen et al. (2015) and Muñoz-Caracuel (2021) are the only studies that have combined EEG and fNIRS in the assessment of sound intensity-dependent changes. However, their fNIRS results did not align with the findings from fMRI studies.

Previous studies have identified a limitation of the fNIRS technique, namely the potential contamination by physiological signals (Caldwell et al. 2016; Tachtsidis and Scholkmann 2016; Zimeo Morais et al. 2018b; Muñoz-Caracuel et al. 2021; Muñoz et al. 2022). Since fNIRS employs infrared light, it can also capture physiological signals such as heart rate, respiration, and blood vasoconstriction. This physiological noise could mask the hemodynamic activity or create false positives, especially when the physiological signals overlap in frequency with the hemodynamic response and cannot be easily filtered. Consequently, careful signal processing is crucial in fNIRS studies to obtain results that accurately reflect cerebral hemodynamic activity. Various techniques have been proposed for signal processing in fNIRS studies. These include regression of short channels to remove extra-cerebral signals (Saager and Berger 2005) and the application of wavelets, principal component analysis (PCA), and filtering, all of which have demonstrated utility. To address this issue, the present report takes the fNIRS data reported by Muñoz-Caracuel et al. (2021), wherein the influence of vasoconstriction at high sound intensity (94.5 dB) on the fNIRS signal was observed, potentially leading to false negative results. The reprocessing of the data includes the use of a PCA filter to extract a component that we hypothesized to be related to the sympathetic process of vascular tone.

Thus, although many studies have focused on the analysis of auditory stimuli, there are still unresolved questions regarding how the brain processes sound, how sound properties, including intensity, are represented neurally, the involvement of neurovascular coupling in this process, and the potential contributions of novel techniques such as fNIRS to addressing these questions. In addition to the characteristics of the hemodynamic response that accompanies the stimulus, an important question is to which degree it is related to the underlying neural activity and, therefore, whether it scales with the intensity of the input stimulus. This study aims to analyze whether fNIRS can be a good tool to detect sound intensity changes in the auditory cortex, which have been related to various psychiatric disorders in ERPs studies. It involves the analysis of two experiments in which stimuli of varying intensities were presented, with the second experiment representing a reanalysis of the data reported in Muñoz-Caracuel et al. (2021). It is hypothesized that the hemodynamic response will scale with the intensity (dB) of the stimulus in the auditory cortex and possibly in the prefrontal cortex. Furthermore, this study could provide evidence to support the neurovascular coupling hypothesis. The potential relevance of this study lies in the fact that a better understanding of neurovascular coupling as a natural function of the healthy brain could become a tool for neurovascular assessment, which could lead to new clinical applications.

## Methods

### Participants

For the first experiment, 33 volunteer subjects (9 males and 24 females, mean  age = 25.21 ± 3.26 years old) participated in the study, and for the second one, 31 subjects (17 males and 14 females, mean age = 25.83 ± 3.90 years old) participated. As a selection criterion, in both experiments, all participants had normal hearing, and none of them had a history of neurological or psychiatric disorders. Before the studies, they were informed about the procedures and the experimental protocol and subsequently signed an informed consent form. The studies follow the rules of the agreements of the latest Declaration of Helsinki for human research (2013) and were approved by the ethics committee of the Junta de Andalucia.

### Procedure

Auditory stimulation was delivered using two Dell A215 speakers placed on each side of the computer monitor and presented using the E-Prime 2.0 software package. For the first experiment, three tones of different intensities (77.9, 84.5, and 89.5 dB) with a duration of 500 ms were presented 54 times. Each tone was followed by a period of silence lasting 14 ± 2 s. The order of the stimuli was randomized for each experimental subject (Supplementary Fig. 1A). In the second experiment, the auditory stimuli consisted of five different tone intensities (70.9, 77.9, 84.5, 89.5, 94.5 dB) presented in trains of eight pure tones, each lasting of 70 ms, with an interstimulus interval of 0.430 ms. Trains of stimuli were followed by 14 ± 2 s of silence and were presented 20 times (Supplementary Fig. 1A). The sound intensity for both experiments was measured and controlled using a sound level meter (Velleman-DVM1326) positioned at the ear location of the participants. The duration of the experiments was approximately 30–35 min, during which participants were instructed to move as little as possible. To enhance tolerance and adherence to the experiment, participants watched a silent movie while the tones were played. The movie served as a distraction, and no active responses were required from the participants.

### Signal acquisition and processing

#### EEG recording

EEG was recorded using Brain Vision V-Amp DC amplifier (Brain Products, Munich, Germany), using active electrodes (ActiCAP), placed in 7 scalp sites (F3 F4 Fz FC1 FC2 FCz Cz) for the first experiment and 11 scalp sites (AFF1, AFF2, FFC1, FFC2, FCC1, FCC2, CZ, CPP1, CPP2, PPO1, PPO2) for the second one (Supplementary Fig. 1B). The eye movements were recorded with 4 electrodes placed: two on the outer edge of each eye to record horizontal movements and the other two above and below the right eye to record vertical movements. Data acquisition was conducted using a BrainVision Recorder 1.20 (Brain Products). The DC amplification gain was set at 20,000 and, no digital filtering was performed during the recording. The sampling rate was 1000 Hz.

First, EEG raw recordings were imported into EEGlab v2021.1 (Swartz Center for Computational Neuroscience; San Diego, CA, USA) and Matlab R2019b (MathWorks, Natick, MA, USA) software packages for processing. For both experiments, EEG data were bandpass filtered from 0.05 to 40 Hz, re-referenced to the right mastoid or averaged (for the second experiment), and segmented into epochs ranging from − 3 to 5 s with a baseline of − 50 to 0 ms. To remove artifacts, an automatic rejection threshold (− 100,100 μV) was applied to all recorded EEG channels. Subjects with less than 95 trials (2 subjects, *n* = 31 for EEG analysis in the first experiment) and less than 80 trials in the second one, were excluded from the analysis (1 subject, *n* = 30 for the second experiment). ERP analysis was performed in the FieldTrip software (Oostenveld et al. 2011) with a time window from − 0.1 to 0.4 s. The ERPs for each stimulus amplitude were calculated through the *ft_timelockanalysis* function*.*

#### Functional near-infrared spectroscopy

The fNIRS signal was recorded with a NIRScoutXP device (NIRx Medical Technologies, Glen Head, NY, USA). In the first experiment, 16 LED sources and 16 detectors placed in temporal areas of both sides of the scalp and the occipital lobe, in locations based on the 10–20 system, were employed. This results in 38 standard channels located 30 mm apart. In addition, to improve the specificity of the fNIRS data targeting cortical activity, 16 short separation channels (8 mm) were added to the fNIRS setup. For the second experiment, 17 LED sources and 38 detectors were employed, and those were located on the frontal and temporal areas of both sides of the scalp, thus obtaining 60 standard channels and 16 short separation channels (Supplementary Fig. 1B). The sampling rate was 3.9063 Hz for the first experiment and 3.67 Hz for the second one. Data acquisition was registered with NIRStar 14.2 software (NIRx Medical Technologies).

Raw fNIRS data were imported into Homer2 (Huppert et al. 2009) and Matlab R2019b (MathWorks Inc., MA, USA) software packages, the pipeline followed for fNIRS signal processing is represented in a flowchart in Supplementary Fig. 2. The *enPruneChannels* function was applied to eliminate noisy channels, removing channels with extreme values (0.03–2.5) or with a high standard deviation (*SNR* = 5 and coefficient of variation = 17). To reduce the motion artifacts in the signal, the function *hmrMotionCorrectionWavele*t was applied, with an Inter Quartile Range (IQR) of 1.5. This function has proven to be quite robust to reduce motion artifacts through wavelet decomposition (Cooper et al. 2012; Molavi and Dumont 2010; Brigadoi et al. 2014). To remove the artifacts, *hmrMotionArtifact* (SDThresh = 15; AMPThresh = 0.7; tmotion = 0.5; tmask = 1.0) was applied, with a time window of − 2 to 10 s around the stimulus (*enStimRejectio*n). To reduce the physiological interference in the signal, a bandpass filter (*hpf* = 0.010, *lpf* = 0.50) was applied. But, taking into account that this filter is not suitable for extracting physiological signals that overlap in frequency with the hemodynamic response, a PCA analysis was performed (*enPCAFilter;* nSV = 1). The number of components to be extracted was verified by employing a spectral power analysis. Supplementary Fig. 3 shows the average PSD of the first component for all subjects, indicating higher power in the LF band between 0.01 and 0.15 Hz. This frequency range has been related to sympathetic vascular control in spectral analysis of the pulse signal (Bernardi et al. 1996; Kiselev and Karavaev 2020; Karavaev et al. 2021). Then, to isolate cortical activity, a regression of the short channel was performed using the *hmrSSR* function. These processing algorithms, as previously stated, aim to enhance signal processing by employing PCA, and filtering of physiological signal before the extraction of components and short channels. This is particularly important in paradigms that could elicit a defensive response, such as vasoconstriction.

Finally, as a last step, hemoglobin concentrations were obtained employing the modified Beer–Lambert law with a differential partial pathlength factor of 6 for 760 nm and 5 for the 850 nm wavelength (Scholkmann et al. 2010). The filtered and processed signal was averaged over a window of − 2 to 13 s, for the first experiment and − 5 to 16 for the second one.

### Statistical analysis

Given the main objective of the present report to analyze the response to different sound intensity levels, a two-way PERMANOVA analysis was performed for both signals (EEG and fNIRS). PERMANOVA was performed in the software PAST (Hammer et al. 2001) with 10,000 permutations, this analysis approach uses permutation testing; therefore, it is not necessary to satisfy the requirements of normality as with ANOVA. The selection of this approach to statistical analysis was mainly motivated by a prior distribution analysis of the fNIRS hemodynamic signal, which did not show a normal distribution.

In the EEG signal, to select the components to analyze, an average of all subjects, electrodes, and stimuli types was performed, as can be seen in Fig. 2A; this approach allows us to select time windows for components analysis in a non-biased manner; thus, the selected components were N1 (100–170 ms) and P2 (190–240 ms for the first experiment and 190–260 ms for the second one), and the N1–P2 peak-to-peak amplitude was also analyzed. Thus, for the PERMANOVA, the factors analyzed were electrode and intensity, for each component (N1, P2, N1–P2 peak-to-peak amplitude), for both experiments, just the central electrodes were analyzed (Supplementary Fig. 1B, electrodes in green), taking into account that in these electrodes is where the auditory ERPs present greater amplitude. Then, a post hoc analysis was performed for the significant factors and corrected for multiple comparisons (false discovery rate (FDR) (Benjamini and Hochberg 1995).

For the fNIRS statistical analysis, the Regions of Interest (ROIs) were selected using the fNIRS Optodes’ Location Decider software (fOLD; Zimeo Morais et al. 2018a). In the first experiment, the channels with the highest specificity for the auditory cortex (Brodmann areas 41–42) and superior temporal gyrus (Brodmann area 22) were selected, comprising the auditory ROI. The primary visual cortex (Brodmann area 17) was chosen for the visual ROI. In the second experiment, in addition to the auditory cortex, the dorsolateral prefrontal cortex (Brodmann area 46) and prefrontal cortex (Brodmann area 9) regions were included. Supplementary Fig. 1B displays the selected channels for the analysis in both experiments. In addition, Supplementary Fig. 1C presents the sensitivity map obtained with Atlasviewer software (Aasted et al. 2015), which allows visualization of the coverage of desired brain regions and their sensitivity. This map was generated specifically for the channels analyzed in each ROI.

Similar to the process for selecting ERPs, an average ROI and intensity were employed to determine an unbiased time window of post-stimulus activity. For the first experiment, a time window of 3.5–7.5 was chosen, considering that the peak of the activity occurred at 5.5 s. In the second experiment, the peak of activity was observed around 6 s, thus a time window of 4–8 s was selected (Supplementary Fig. 4). Prior to the statistical analysis, subjects with extreme values (more than 25% artifact values) were excluded using the *isoutlier* function. Therefore, the fNIRS statistical analysis included 31 subjects for the first experiment and 29 subjects for the second experiment.

The selected channels for each region were collapsed into the left auditory cortex, right auditory cortex, and visual cortex (*ROI* = 3) for the first experiment. In the second experiment, the left auditory cortex, right auditory cortex, left dorsolateral cortex, right dorsolateral cortex, and prefrontal cortex (*ROI* = 5) were considered. The factors analyzed were ROIs and intensity, for each type of hemoglobin (HbO, HbR, and HbT). A post hoc analysis with the significant factors was performed and then corrected using the false discovery rate (FDR). For the first experiment, the visual channel was used as a control. Comparisons were made with the baseline for HbO and HbR in each ROI (collapsed intensity) for each hemoglobin type. In addition, to assess neurovascular coupling a Spearman correlation (FDR corrected) was performed between the P2 and N1 amplitudes for the electrode with the highest activity (to reduce data dimensionality) and the fNIRS intensity in HbO, HbR, and HbT for each ROI.

## Results

### ERPs

**Fig. 1:**
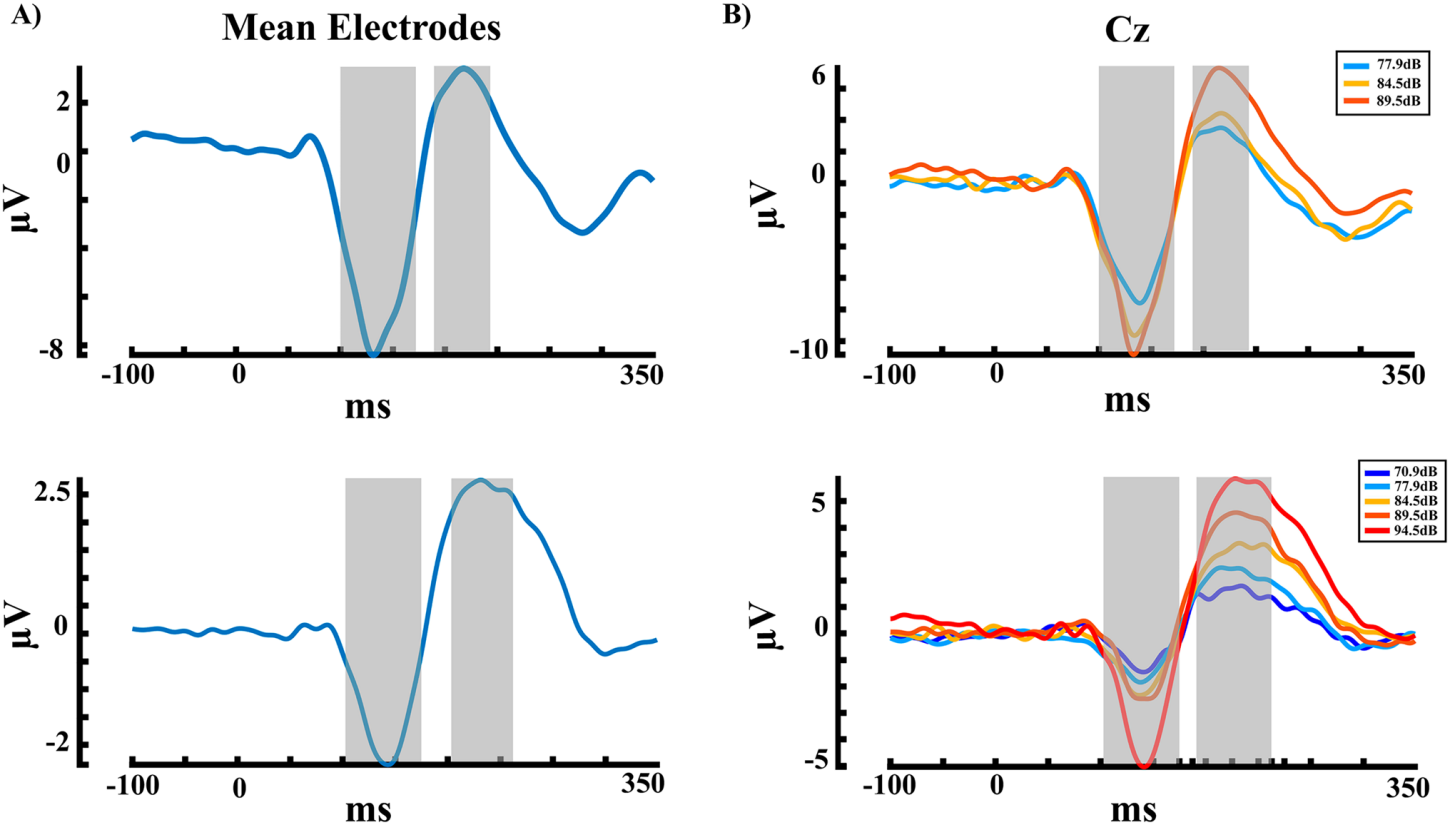
**A** Auditory ERPs for the mean of the electrodes and the mean of the intensity. **B** Auditory ERPs for the presented auditory intensities in the Cz electrode. Above for the first experiment and below for the second experiment. The time windows selected for statistical analysis are marked in grey

**Fig. 2:**
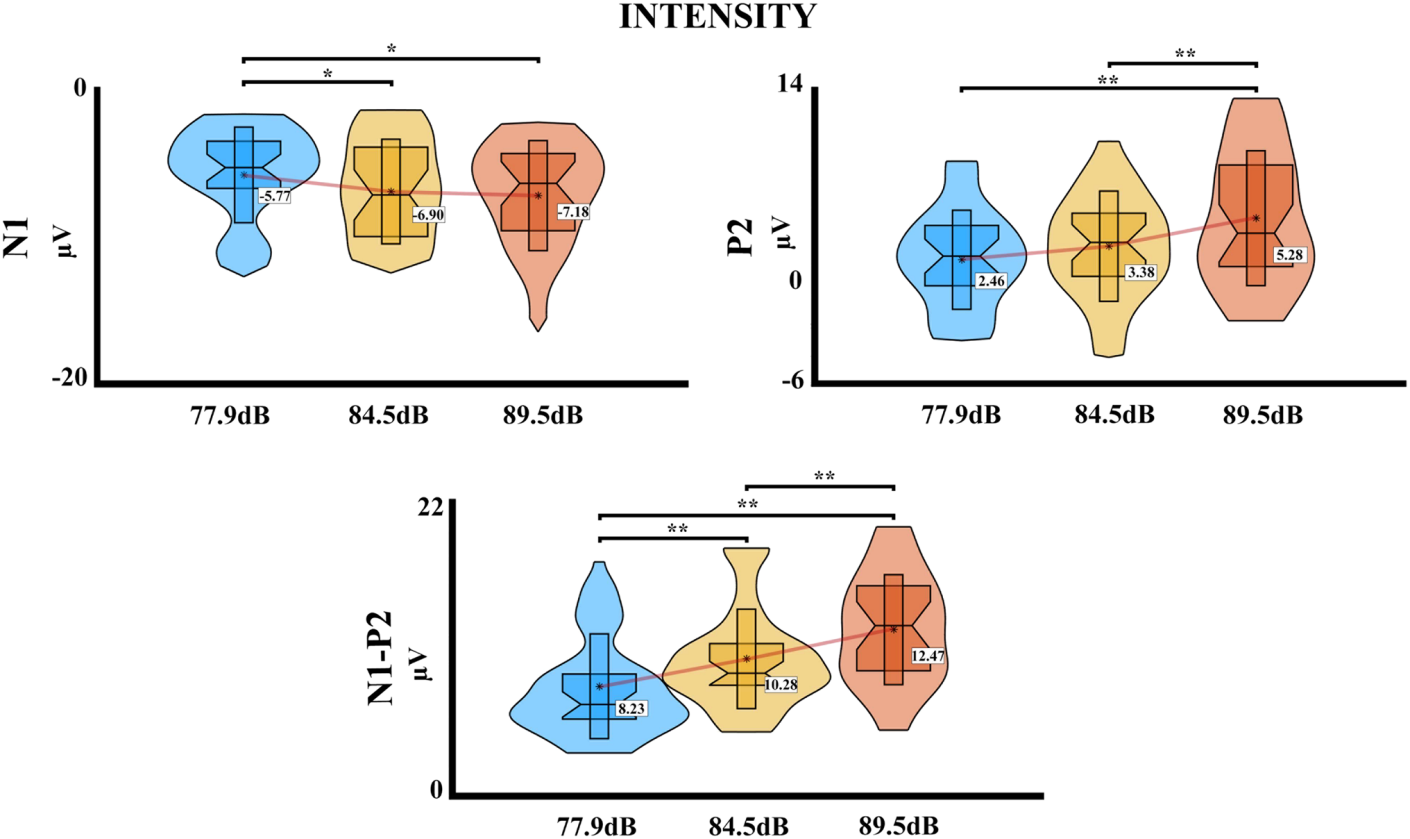
Violin boxplot of the ERPs results with significant effects for the first experiment with three intensities. The mean is represented for each intensity with a red line; *p ≤ 0.05; **p ≤ 0.001

Figure 1B shows the intensities dependent response for the ERPs, and the time windows selected for the analysis in both experiments. For the first experiment with three intensities, the PERMANOVA for the N1 component shows an effect of intensity (*pseudo*-F = 5.42, *η*^2^ = 0.029, *p* = 0.005). Effects of electrode and interaction (intensity*electrode) were not significant (all *p* > 0.05). Post hoc FDR corrected for the intensity showed significant differences between the intensity 1 (77.9 dB) with the intensities 2 (84.5 dB) and 3 (89.5 dB) (*p* = 0.006, Int1 > Int3; *p* = 0.017, Int1 > Int2). For the P2 component, the PERMANOVA shows an effect of intensity (*pseudo*-F = 15.733, *η*^2^ = 0.079, *p* < 0.001), and the effects of electrode and interaction (intensity*electrode) were not significant (all *p* > 0.05). The FDR-corrected analysis showed significant differences for the intensity 3 (89.5 dB) with the intensities 1 (77.9 dB) and 2 (84.5 dB) (*p* < 0.001, Int1 < Int3; *p* < 0.001, Int2 < Int3). The N1–P2 peak-to-peak amplitude PERMANOVA results show an effect of intensity (*pseudo*-F = 34.44,* η*^2^ = 0.158, *p* < 0.001), and effects of electrode and interaction (intensity*electrode) were not significant (all *p* > 0.05). FDR-corrected post hoc analysis showed a significant difference of the intensity 1 (77.9 dB) with the intensities 2 (84.5 dB) and 3 (89.5 dB) (*p* < 0.001, Int1 < Int3; *p* < 0.001, Int1 < Int2), and also between the intensity 2 (84.5 dB) and 3 (89.5 dB) (p = 0.001, Int2 < Int3). The results for the comparisons and the distribution of the data are shown in Fig. 2.

**Fig. 3:**
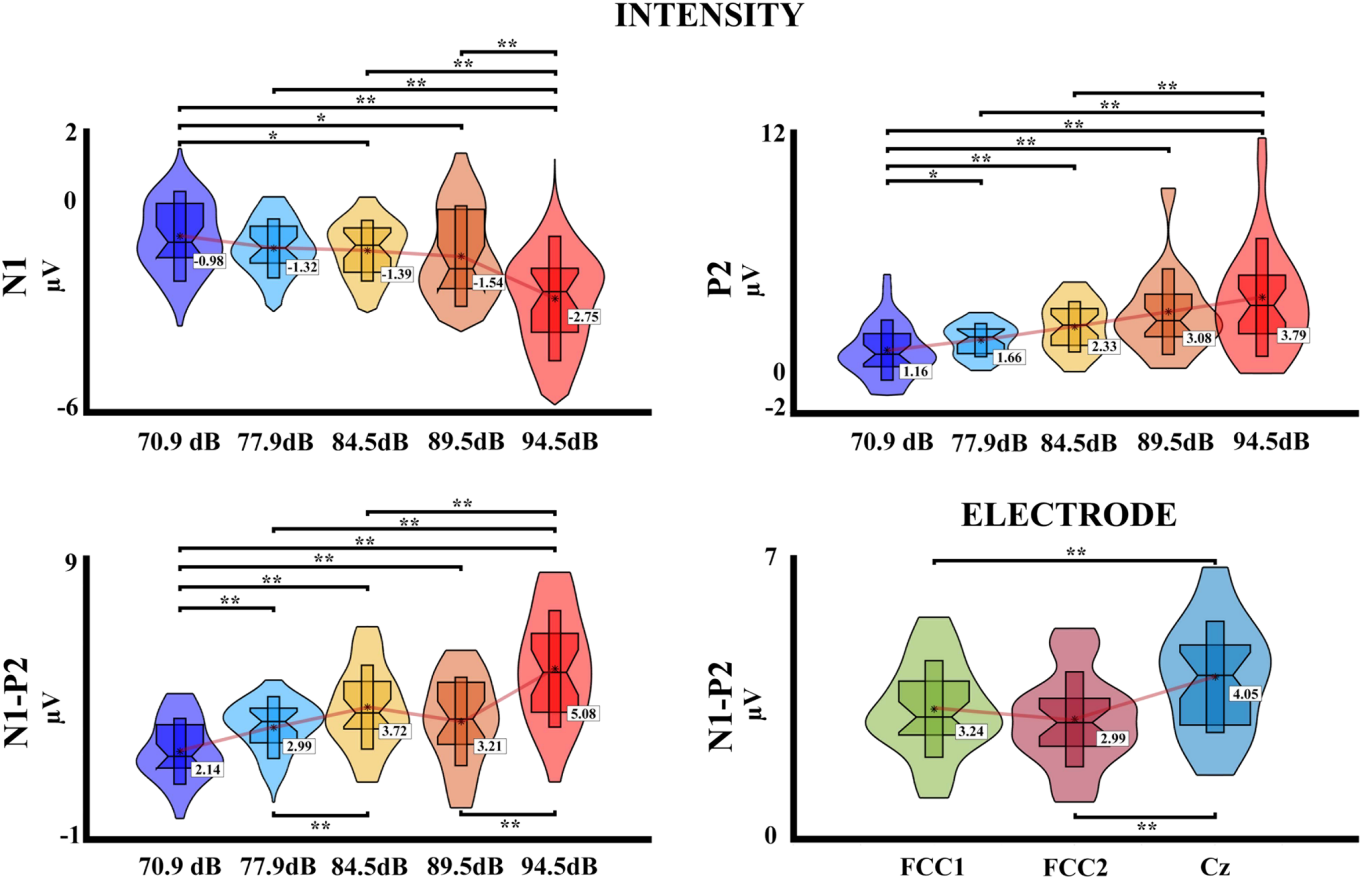
Violin boxplots of the ERPs results with significant effects for the second experiment with five intensities. The mean is represented for each intensity or electrode with a red line; *p ≤ 0.05; **p ≤ 0.001

For the second experiment with five intensities, the PERMANOVA showed an effect of intensity for the N1 component (*pseudo*-F = 21.10, *η*^2^ = 0.160, *p* < 0.001), and the effects of electrode and interaction (intensity*electrode) were not significant (all *p* > 0.05). The post hoc FDR-corrected analysis showed a significant difference between the intensity 1 (70.9 dB) with the intensities 3 (84.5 dB), 4 (89.5 dB), and 5 (94.5 dB) (*p* = 0.035, Int1 > Int3; *p* = 0.023, Int1 > Int4; *p* < 0.001, Int1 > Int5), and also for the intensity 5 with the intensities 2 (77.9 dB), 3 (84.5 dB), and 4 (89.5 dB) (*p* < 0.001, Int2 > Int5; *p* < 0.001, Int3 > Int5; *p* < 0.001, Int4 > Int5). For the P2 component, the PERMANOVA shows an effect for both electrode (*pseudo*-F = 16.44, *η*^2^ = 0.057, *p* < 0.001) and intensity (*pseudo*-F = 25.05, *η*^2^ = 0.174, *p* < 0.001), and the effect of interaction (intensity*electrode) was not significant (*p* > 0.05). The post hoc analysis for the electrodes showed a significant difference between the electrode Cz with the electrodes FCC1 and FCC2 (*p* < 0.001, FCC1 < Cz; *p* < 0.001, FCC2 < Cz). For the intensity, the FDR-corrected post hoc analysis showed significant differences between the intensity 1 with the intensities 2 (77.9 dB), 3 (84.5 dB), 4 (89.5 dB), and 5 (94.5 dB) (*p* = 0.018, Int1 < Int2; p < 0.001, Int1 < Int3; *p* < 0.001, Int1 < Int4; *p* < 0.001, Int1 < Int5), and also between the intensity 5 with the intensities 2 (77.9 dB), and 3 (84.5 dB) (p < 0.001, Int2 < Int5; *p* < 0.001, Int3 < Int5). Finally, for the N1–P2 peak-to-peak difference, the PERMANOVA shows effect of electrode (*pseudo*-F = 16.12, *η*^2^ = 0.052, *p* < 0.001) and intensity (*pseudo*-F = 37.49, *η*^2^ = 0.240, *p* < 0.001), and the effect of interaction (intensity*electrode) was not significant (*p* > 0.05). The post hoc analysis for the electrodes, similar to P2 results, shows a significant difference between the electrode Cz with the electrodes FCC1 and FCC2 (*p* < 0.001, FCC1 < Cz; *p* < 0.001, FCC2 < Cz) For the intensity, the FDR-corrected post hoc analysis showed significant differences between the intensity 1 with the intensities 2 (77.9 dB), 3 (84.5 dB), 4 (89.5 dB), and 5 (94.5 dB) (*p* < 0.001, Int1 < Int2; *p* < 0.001, Int1 < Int3; *p* < 0.001, Int1 < Int4; *p* < 0.001, Int1 < Int5), the intensity 5 with the intensities 2 (77.9 dB), 3 (84.5 dB) and 4 (89.5 dB) (*p* < 0.001, Int2 < Int5; *p* < 0.001, Int3 < Int5, *p* < 0.001, Int4 < Int5), and lastly between the intensities 2 (77.9 dB) and 3 (84.5 dB) (*p* = 0.001, Int2 < Int3). The post hoc comparisons and the data distribution are shown in Fig. 3.

### Hemodynamic response

**Fig. 4:**
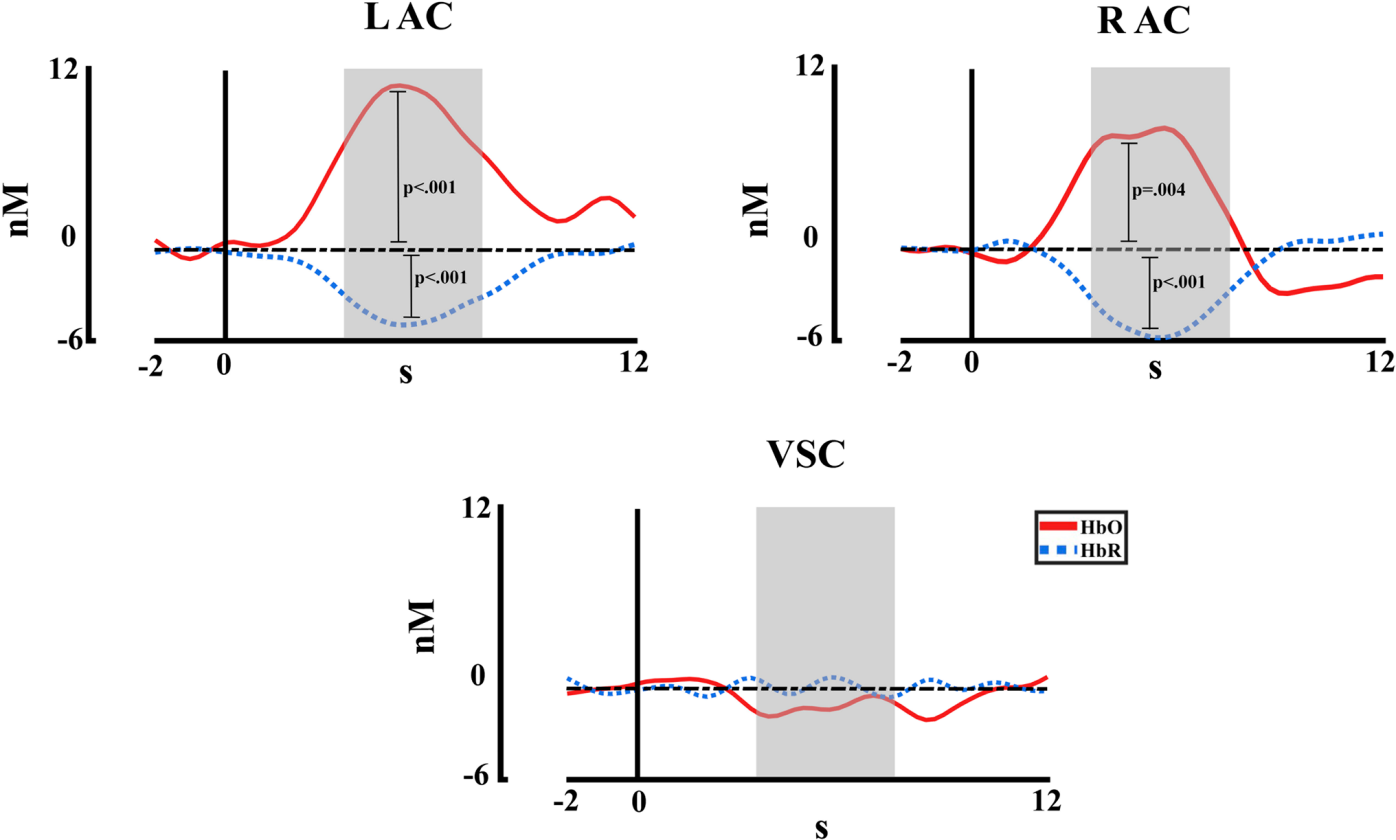
fNIRS concentration changes averaged across intensities for HbO and HbR in each region of interest. R AC (right auditory cortex), L AC (left auditory cortex), VSC (Visual cortex). The statistical significance of comparison with baseline is represented. The time window selected for statistical analysis is marked in grey

For the analysis of the hemodynamic response in the first experiment, first, a t-test comparison to the baseline was performed for each ROI. The analysis was conducted with the intensities averaged (Fig. 4), to analyze the activation level of the ROIs to the auditory stimulation. The results showed for the HbO chromophore a significant difference for the left and right auditory cortices compared to baseline (L AC *p* < 0.001, *t* = 4.21; R AC *p* = 0.007, *t* = 2.86), and similarly, for the HbR chromophore a significant difference for the left and right auditory cortices compared to baseline (L AC *p* < 0.001, *t* = − 4.99; R AC *p* < 0.001, *t* = − 3.92). The t-values showed the expected values for a hemodynamic canonical response, i.e., positive amplitude values for HbO and negative values for HbR, in both auditory cortices. The expected lack of significance for the visual ROI was also obtained.

**Fig. 5:**
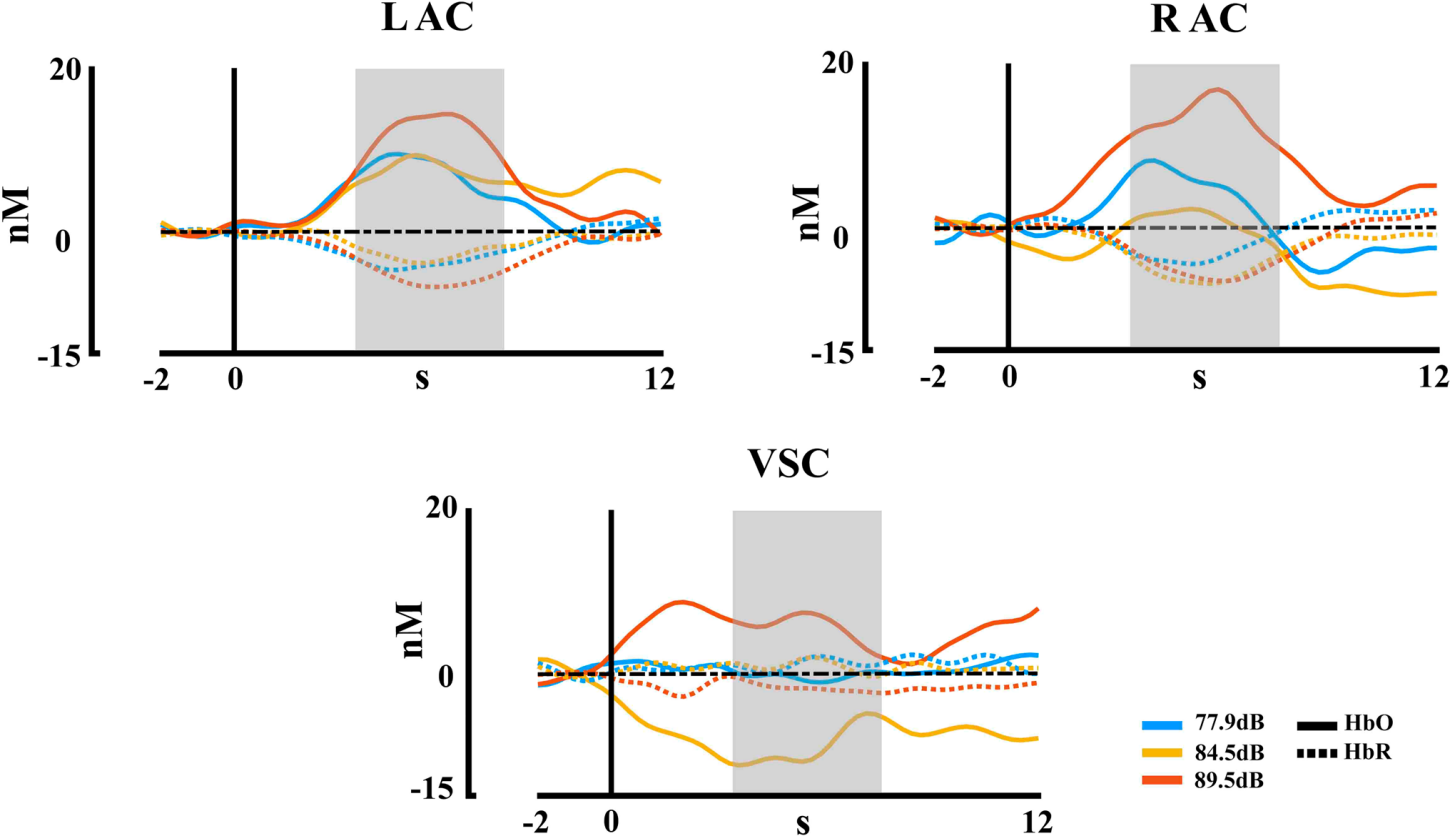
fNIRS concentration changes for HbO and HbR for each stimulation intensity in each region of interest of the first experiment. R AC (right auditory cortex), L AC (left auditory cortex), VSC (Visual cortex). HbR is shown with dashed lines. The time window selected for statistical analysis is marked in grey

**Fig. 6:**
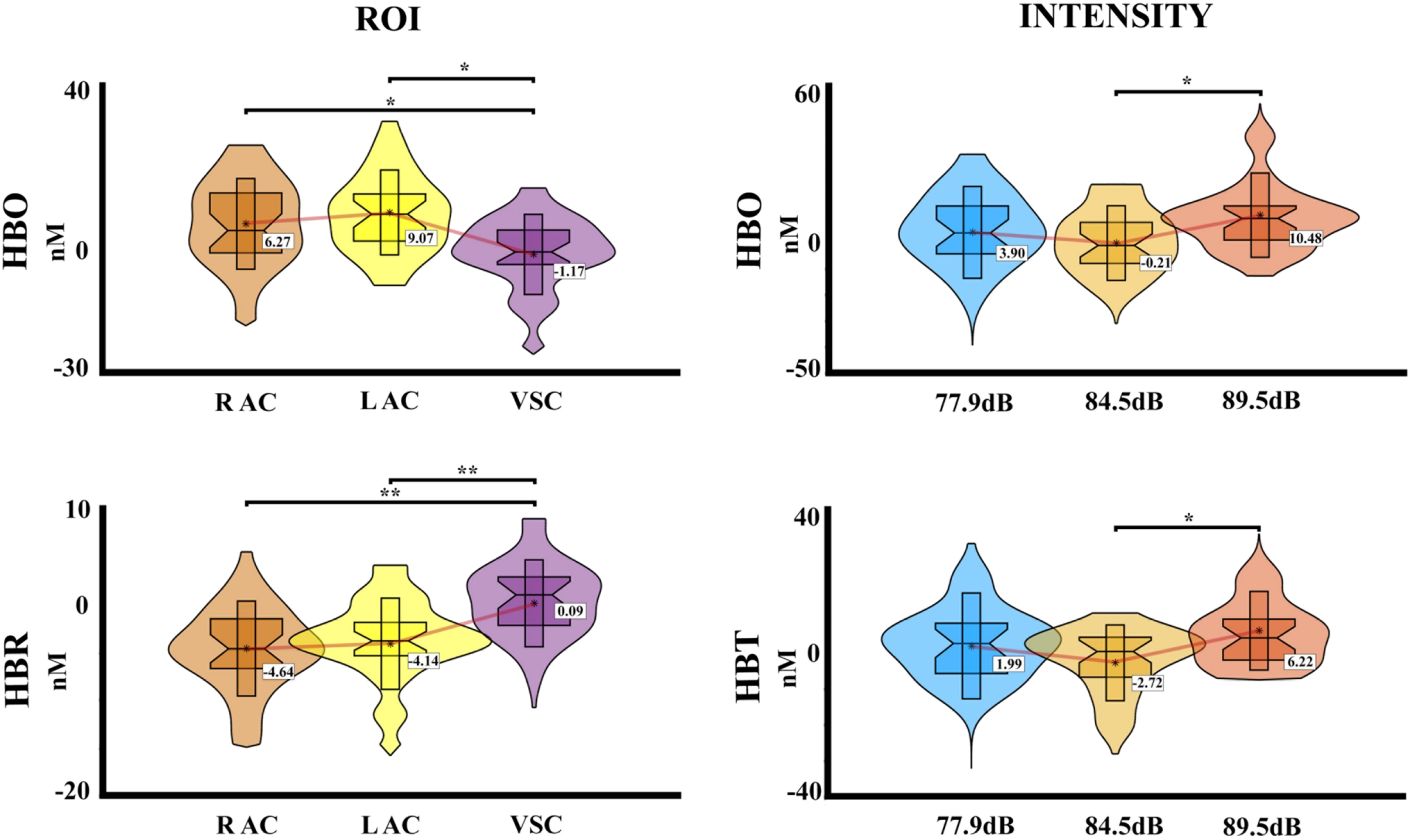
Violin boxplot for the significant effects of the fNIRS statistical results for each hemoglobin type (HbO, HbR, and HbT) in the first experiment with three intensities. Mean is represented for each intensity and ROI with a red line. R AC (right auditory cortex), L AC (left auditory cortex), VSC (Visual cortex); *p ≤ 0.05; **p ≤ 0.001

Figure 5 shows the hemodynamic response recorded with fNIRS for HbO and HbR chromophores in the different ROIs, in response to the three auditory stimuli delivered. The PERMANOVA analysis for HbO concentrations shows an effect for the ROIs factor (*pseudo*-F = 5.02, *η*^2^ = 0.034, *p* = 0.007) and intensity factor (*pseudo*-F = 5.22, *η*^2^ = 0.036, *p* = 0.006), and the effect of interaction (intensity*ROI) was not significant (*p* > 0.05). The post hoc analysis FDR corrected for the ROI showed a significant difference between the visual cortex and the right and left auditory cortices (*p* = 0.023, VSC < R AC; *p* = 0.002, VSC < L AC). For the intensity, the post hoc analysis showed a significant difference between intensity 2 (77.9 dB) with the intensity 3 (84.5 dB) (*p* = 0.012, Int2 < Int3). For the HbR concentrations, the PERMANOVA showed an effect of ROIs (*pseudo*-F = 7.86,* η*^2^ = 0.054, *p* < 0.001), and the effects of intensity and interaction (intensity*ROI) were not significant (all *p* > 0.05). The post hoc analysis FDR corrected showed a significant difference between the visual cortex with the right and left auditory cortices (*p* < 0.001, VSC > R AC; *p* = 0.001, VSC > L AC). The PERMANOVA of the HbT concentrations showed an effect of intensity (*pseudo*-F = 5.05, *η*^2^ = 0.035, *p* = 0.006), and the effects of ROI and interaction (intensity*ROI) were not significant (all *p* > 0.05). The post hoc analysis FDR corrected showed a significant difference between the intensity 2 (77.9 dB) and 3 (84.5 dB) (p = 0.007, Int2 < Int3). The summarized results and its distribution are shown in Fig. 6.

**Fig. 7:**
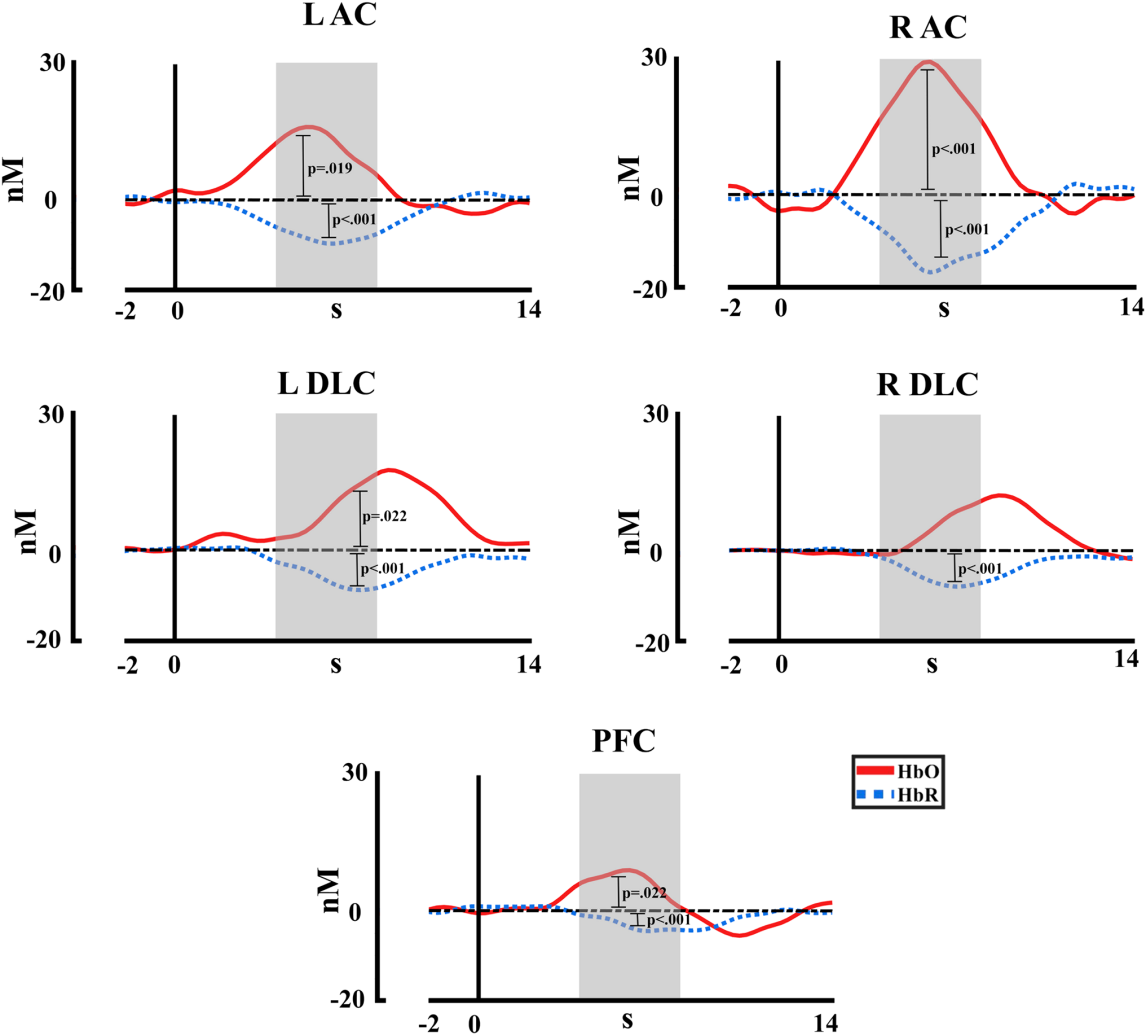
fNIRS concentration changes averaged across intensities for HbO and HbR in each region of interest. R AC (right auditory cortex), L AC (left auditory cortex), L DLC (left dorsolateral cortex), R DLC (right dorsolateral cortex), PFC (prefrontal cortex). The statistical significance of comparison with baseline is represented. The time window selected for statistical analysis is marked in grey

For the second experiment, similar to the first experiment, a t-test comparison with baseline (FDR corrected) was performed by each ROI with the intensities averaged (Fig. 7). The results showed a significant difference for the HbO chromophore for the left auditory cortex, right auditory cortex, left dorsolateral cortex and prefrontal cortex (L AC *p* = 0.019, *t* = 2.87; R AC *p* < 0.001, *t* = 7.10; L DLC *p* = 0.022, *t* = 2.58; PFC *p* = 0.022, *t* = 2.51). For HbR, all ROIs showed significant differences compared to baseline (L AC *p* < 0.001, *t* = − 5.25; R AC *p* < 0.001, *t* = − 6.79; L DLC *p* < 0.001, *t* = − 4.69; R DLC *p* < 0.001, *t* = − 4.277; PFC *p* < 0.001, *t* = − 4.56). Also, similar to the first experiment, positive t-values were obtained for HbO and negative t-values for HbR. As can be seen in Fig. 7, the lack of effect in the right dorsolateral cortex for HbO concentrations could be due to the delayed spike shift in the dorsolateral cortex, which seems to occur with a delay compared to the spike in the auditory and prefrontal cortices.

**Fig. 8:**
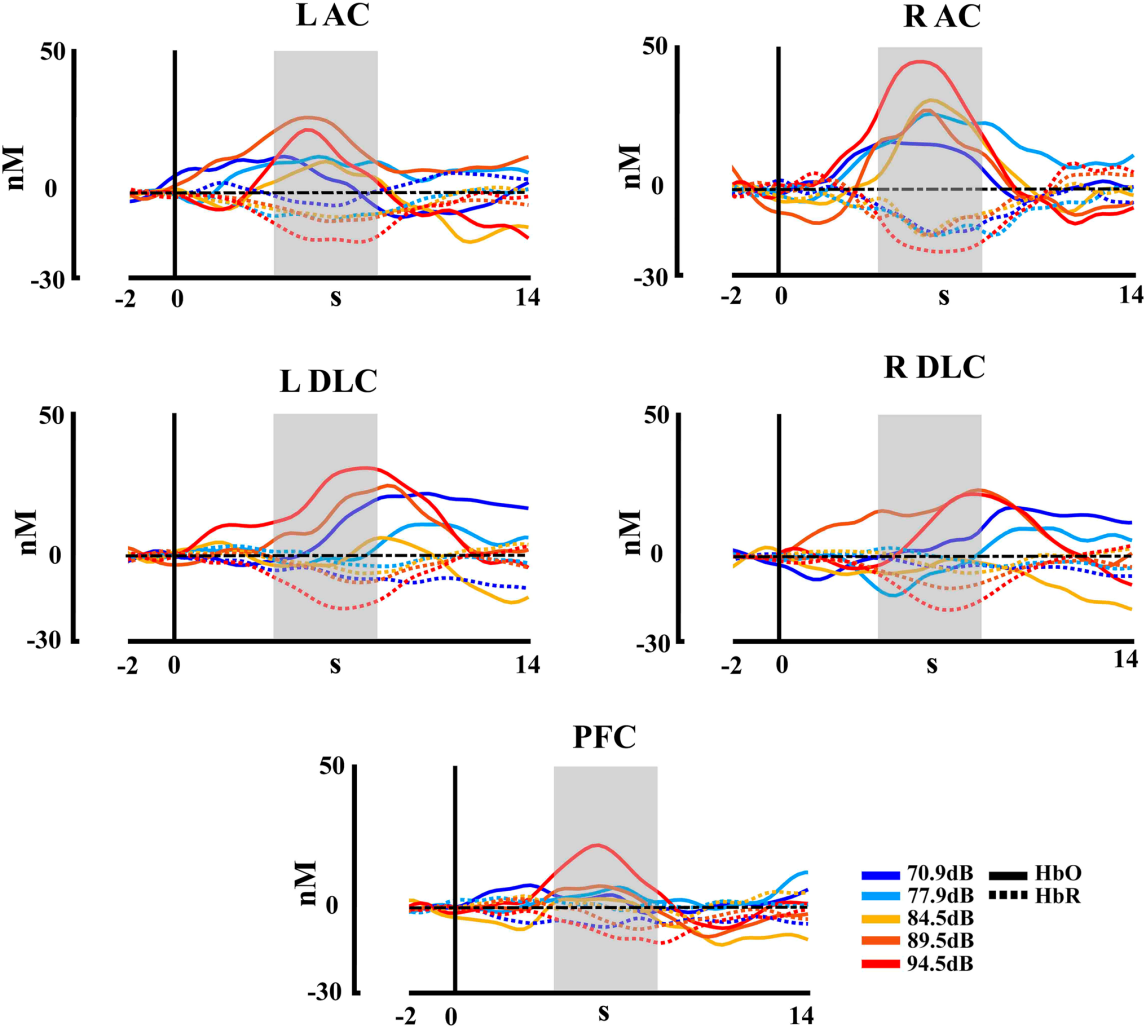
fNIRS concentration changes for HbO and HbR for each stimulation intensity in each region of interest of the second experiment. R AC (right auditory cortex), L AC (left auditory cortex), L DLC (left dorsolateral cortex), R DLC (right dorsolateral cortex), PFC (prefrontal cortex). HbR is shown with dashed lines

**Fig. 9:**
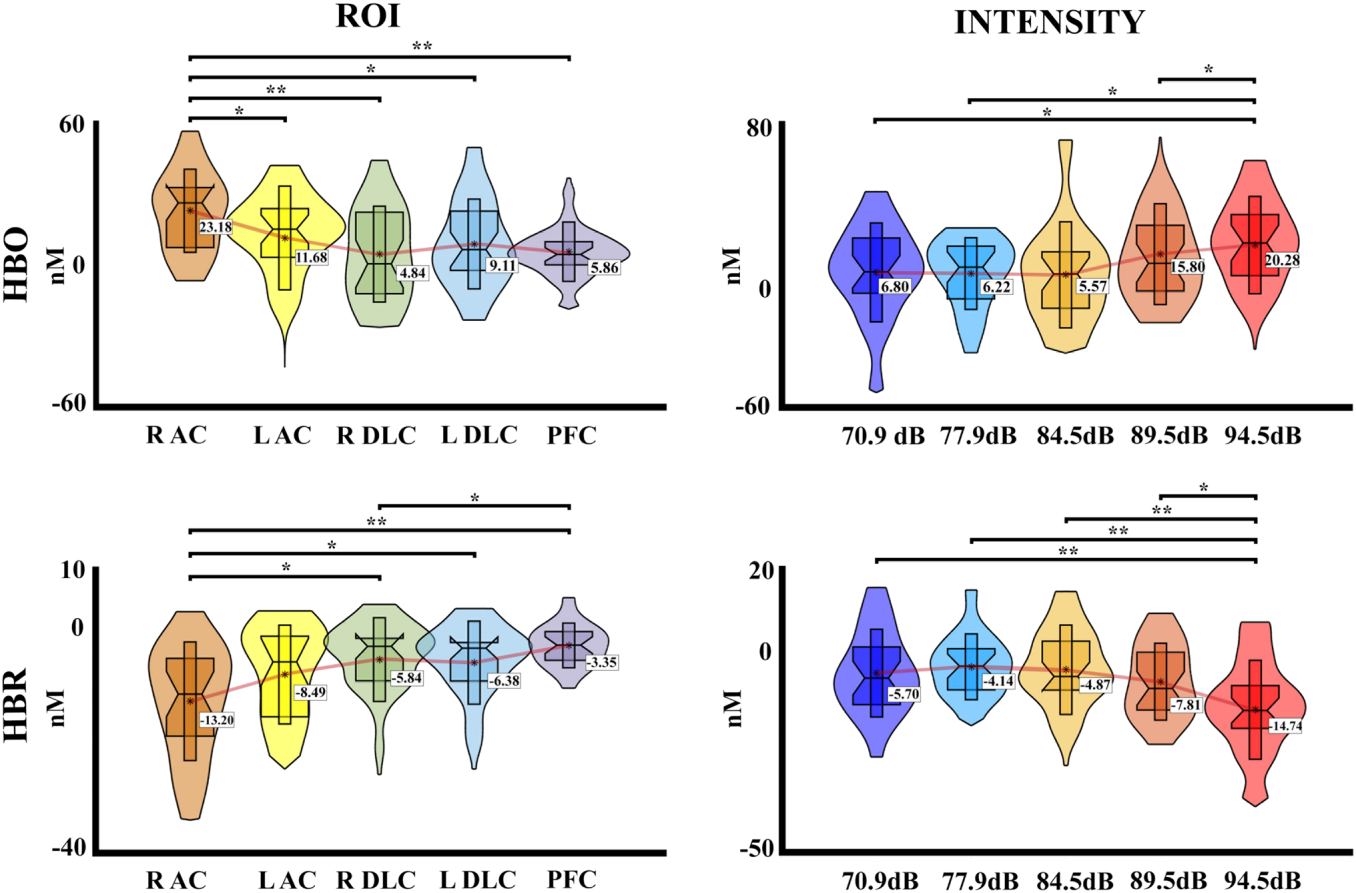
Violin boxplot for the significant effects of the fNIRS statistical results for each hemoglobin type (HbO, HbR) in the second experiment with five intensities. Mean is represented for each intensity and ROI with a red line. R AC (right auditory cortex), L AC (left auditory cortex), L DLC (left dorsolateral cortex), R DLC (right dorsolateral cortex), PFC (prefrontal cortex); *p ≤ 0.05; **p ≤  0.001

Figure 8 shows the hemodynamic response for HbO and HbR chromophores in the five ROIs, in response to the five auditory stimuli presented (70.9 dB, 77.9 dB, 84.5 dB, 89.5 dB, and 94.5 dB). The PERMANOVA for HbO concentrations shows an effect of ROIs (*pseudo*-F = 5.12, *η*^2^ = 0.027, *p* < 0.001) and intensity factors (*pseudo*-F = 4.23, *η*^2^ = 0.023, *p* = 0.003), and the effect of interaction (intensity*ROI) was not significant (*p* > 0.05). For the ROIS the post hoc analysis FDR corrected showed a significant difference between the right auditory cortex with the left auditory cortex, the right dorsolateral cortex, the left dorsolateral cortex, and the prefrontal cortex (*p* = 0.027, R AC > L AC; *p* = 0.001, R AC > R DLC; *p* = 0.018, R AC > L DLC; *p* = 0.001, R AC > PFC). For the intensity a significant difference between the intensity 5 (94.5 dB) with the intensities 1 (70.9 dB) 2 (77.9 dB), and 3 (84.5 dB) (*p* = 0.015, Int1 < Int5; *p* = 0.014, Int2 < Int5; *p* = 0.014, Int3 < Int5). For the HbR concentrations, the PERMANOVA shows an effect of ROIs (*pseudo*-F = 6.74, *η*^2^ = 0.035, *p* < 0.001) and intensity (*pseudo*-F = 9.13, *η*^2^ = 0.047, *p* < 0.001), and the effect interaction (intensity*ROI) was not significant (*p* > 0.05). The post hoc analysis FDR corrected for the ROI showed a significant difference between the right auditory cortex with the right dorsolateral cortex, the left dorsolateral cortex, and the prefrontal cortex (*p* = 0.005, R AC < R DLC; *p* = 0.005, R AC < L DLC; *p* = 0.001, R AC < PFC), and also for the left auditory cortex with the prefrontal cortex (*p* = 0.016, L AC < PFC). For the intensity, the post hoc analysis showed significant differences between the intensity 5 with the intensities 1 (70.9 dB) 2 (77.9 dB), 3 (84.5 dB), and 4 (89.5 dB) (*p* < 0.001, Int1 > Int5; *p* < 0.001, Int2 > Int5; *p* < 0.001, Int3 > Int5; *p* = 0.004, Int4 > Int5). The significant effects and the data distribution are shown in Fig. 9. The HbT showed no effect for ROIs, intensity, or interaction (all *p* > 0.05).

**Fig. 10:**
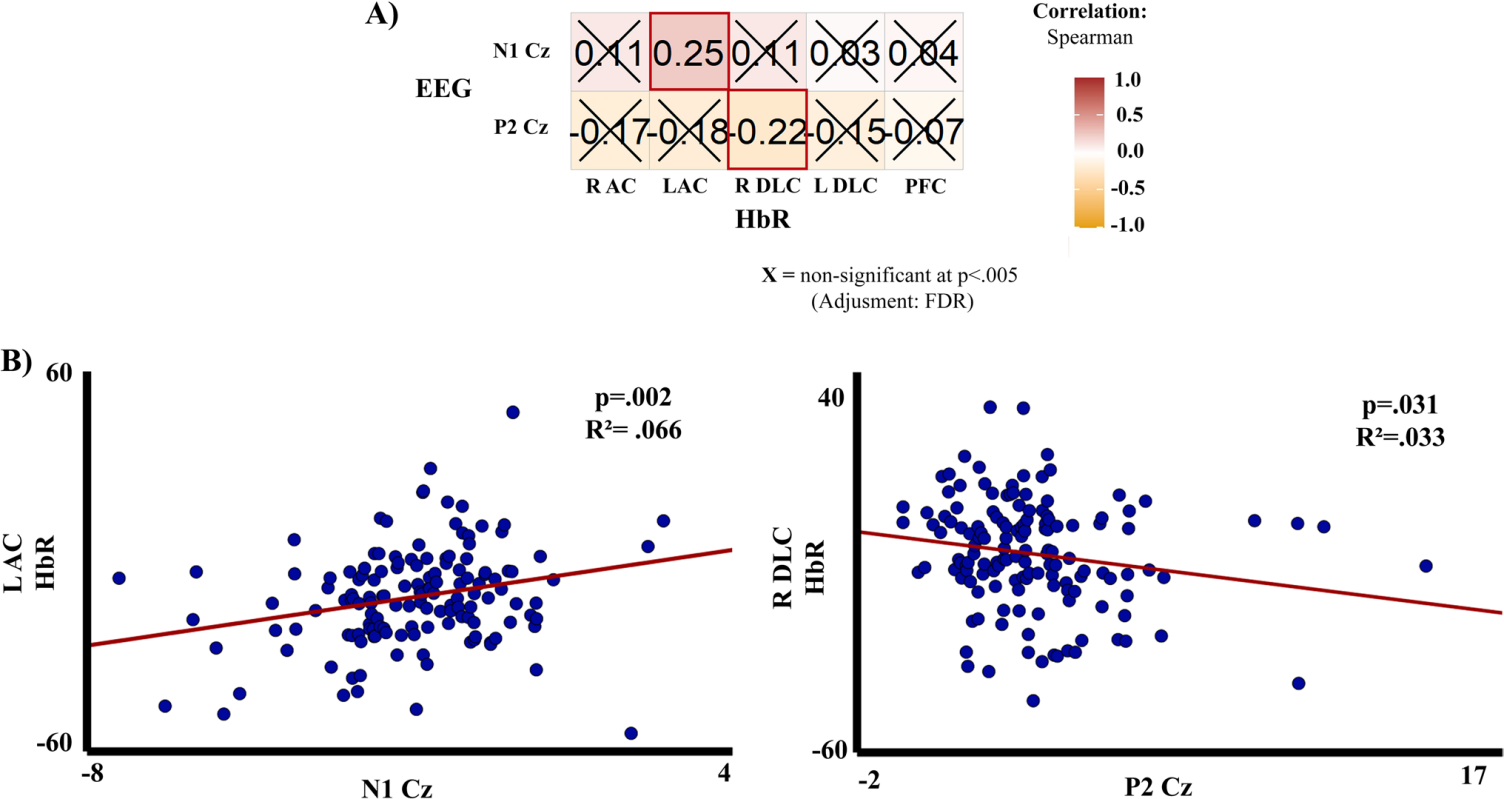
**A** Spearman correlation (FDR corrected) of HbR and the N1 and P2 amplitudes for the Cz electrode, for the second experiment. **B** Linear regression for the significant correlations of N1 and P2 with fNIRS HbR concentrations. R AC (right auditory cortex), L AC (left auditory cortex), L DLC (left dorsolateral cortex), R DLC (right dorsolateral cortex), PFC (prefrontal cortex). Correlation exported from R with the function *ggstatsplots* (Patil 2021)

For the Spearman correlation analysis in the first experiment, given that PERMANOVA did not show an electrode effect, the correlation was performed between the N1 and P2 amplitudes for the mean of the EEG central electrodes and the HbO, HbR, and HbT (independently) by ROI, and the results did not show any significant correlation. For the second experiment, the Spearman correlation FDR corrected was performed between the N1 and P2 amplitudes for the Cz electrode and the HbO, HbR, and HbT (independently) by ROI. The selection of the Cz electrode for the correlation was motivated by the greater amplitude of this electrode compared to FCC1 and FCC2 electrodes, shown in the PERMANOVA. The results did not show a significant correlation for HbO and HbT, just for the HbR chromophore significant correlations FDR corrected were found between the left auditory cortex for N1 amplitude (*p* = 0.031) and for the right dorsolateral cortex with P2 amplitude (*p* = 0.040), Fig. 10 shows the Spearman correlation matrix and the linear regression, being as expected positive for N1 (*p* = 0.002) and negative for P2 (*p* = 0.031).

## Discussion

The present study analyzed the effect of sound intensity modulation on the auditory and prefrontal cortices using fNIRS, and EEG, as a way to find the neurovascular coupling between both measures. The expected modulation was observed in the amplitude of the auditory ERPs N1, P2, and N1–P2 peak-to-peak amplitude in the EEG signal. Furthermore, both experiments revealed an intensity effect on the hemodynamic response measured with fNIRS in the auditory cortex, and in the second experiment, in the prefrontal cortices as well. The PERMANOVA analysis and the significant findings in the comparison with baseline analysis, revealed an effect of ROIs in the auditory and prefrontal channels, with an increase in the amplitude of the HbO and a decrease in the amplitude of the HbR, supporting the presence of a hemodynamic response to the auditory stimulation. In addition, Spearman correlations suggested a potential contribution of the auditory cortex to the generation of N1 and the dorsolateral cortex to P2. Together our results highlight the sensitivity of fNIRS in assessing auditory cortex activation and modulation. Therefore, this study contributes to expanding the understanding of the applicability of spectroscopy in the investigation of auditory paradigms.

The results of the present study showed intensity modulation-dependent amplitude changes for N1, P2, and the N1–P2 peak-to-peak amplitude in response to acoustic stimuli of three and five different intensities (77.9 dB, 84.5 dB, and 89.5 dB; 70.9 dB, 77.9 dB, 84.5 dB, 89.5 dB, and 94.5 dB). These findings are consistent with previous studies (Hegerl and Juckel 1993; Hegerl et al. 1994; Dierks et al. 1999; Muñoz-Caracuel et al. 2021) that reported an increase in amplitude in N1 and P2 components with increasing intensity levels, as well as an increase in the N1–P2 difference. The results suggest that the employed stimulation paradigm is suitable for studying intensity-dependent amplitude changes, aligning with the existing literature that has consistently observed similar effects in ERPs.

The fNIRS results revealed a significant effect of intensity in both experiments. Although no interaction effect between intensity and ROI was found, the comparison with baseline demonstrated activation in the auditory and prefrontal cortex in the second experiment. This activation is characterized by increased levels of HbO and decreased levels of HbR compared to baseline. The observed intensity effect in the fNIRS signal supports is consistent with previous fMRI studies that have identified the primary auditory cortex as the source of the intensity effect (Hall et al 2001; Hart et al. 2003; Langers et al. 2007; Röhl and Uppenkamp 2012). The activation in the prefrontal cortices reported in the second experiment aligns with findings from EEG source analysis studies (Giard et al. 1994; Gallinat et al. 2002; Zhang et al. 2011), and fMRI studies by Bauernfeind et al. (2018) and Neuner et al (2014) that reported activation in frontal regions such as the medial temporal gyrus, orbital, triangular, and opercular part. The authors have related this activation to the associative function of this cortex in the retrieval and rehearsal of auditory information, and the evaluation of aversive auditory stimuli. The correlation found in the HbR chromophore with N1 and P2 amplitudes could suggest a possible contribution for these ERPs. For N1 in the auditory cortex, supporting previous studies (Näätänen and Picton 1987; Woods 1995). Furthermore, the correlation between P2 and dorsolateral fNIRS activity provided evidence for a prefrontal contribution to the P2 component. However, the neural source of P2 is still a subject of debate in the literature (Paiva et al. 2016).

The indirect nature of fNIRS and its limited depth coverage compared to fMRI, along with the influence of physiological factors on cerebral hemodynamics, may contribute to the contradictory findings observed in the literature (Chen et al. 2015; Muñoz-Caracuel et al. 2021; Muñoz et al. 2022). According to Mulert (2010), the synchronous activity of a small number of neurons can lead to detectable changes in EEG signals, but the corresponding hemodynamic changes might not be strong enough to be distinguished from the brain's baseline activity (resting state) using statistical analysis, suggesting that the hemodynamic changes are subtlest compared to EEG signal. Moreover, the study of cerebral hemodynamics is influenced by physiological factors such as vascular compliance and blood perfusion, which can impact the presence of a hemodynamic response to stimuli. Animal studies (Schei et al. 2012) have shown that sustained neural activity can decrease vascular distensibility, potentially limiting vascular responsiveness and local blood perfusion. Consequently, conditions that stress the cerebral vasculature could further reduce vascular distensibility, making it more difficult to find the neurovascular coupling response between the electrical and hemodynamic signal.

In humans, vascular responses to stress conditions have been extensively studied in autonomic and peripheral measurements. High-intensity sounds have been shown to induce vasoconstriction, as analyzed by measures such as Pulse transit time (PTT) and/or Power spectral density (PSD) of the pulse signal, both vasoconstriction indicators (Franco et al. 2002; Galland et al. 2007; Muñoz et al. 2022). This issue becomes particularly relevant in studies where the defense reflex may mask the hemodynamic signals, as in the present report when high intensities are employed, and how has been hypothesized by previous studies of our laboratory (Muñoz-Caracuel et al 2021; Muñoz et al. 2022). Thus, in the present report, this limitation was tried to avoid by careful processing of the signal with PCA and filtering of physiological signals after the extraction of the components and short channels signal. It is worth noting that this improvement in the processing pipeline is supported for the recovering of the hemodynamic signal in the fNIRS dataset of the second experiment, considering that in Muñoz-Caracuel et al. (2021) a vasoconstriction process was reported with a decrease in the HbO concentrations to the high intensity (94.5 dB). Another technical challenge with hemodynamics signals, as proposed by Steinmetzger et al. (2020) is the phenomenon of blood-stealing that could potentially mask the effect of intensity in the auditory cortex. The authors suggest that non-active exhibit a negative HbO response pattern, similar to negative BOLD responses, which reflects the inhibition of irrelevant neural populations. Consequently, these negative HbO responses in channels near the active region could obscure the effects of the cortex when averaging across ROIs. Similarly, Shader et al. (2021) found deactivation patterns in adjacent subregions of the superior occipital gyrus as well as cuneus in response to the visual-only speech. To mitigate this effect, we focused our analysis on channels with high specificity for the primary auditory cortex and superior temporal gyrus, which in previous fMRI studies have been shown to have intensity modulation dependence (Thaerig et al. 2008; Neuner et al. 2014). In conjunction with specific channel selection, appropriate filtering, and extraction of physiological components, we believe that the hemodynamic signal can be properly extracted from the fNIRS signal to be studied in auditory paradigms that have high sound intensities.

As for limitations, it is important to acknowledge that although an intensity effect was observed in the fNIRS signal in the present study, the statistical results when compared to the ERPs were less robust. This could be attributed to the small difference in decibel levels between the stimuli, which may not have been sufficient to produce significant changes in the hemodynamic response. Therefore, future research on intensity-dependent amplitude changes, particularly with fNIRS, is recommended to utilize stimuli with a larger decibel gap to enhance the detectability of the hemodynamic response. Furthermore, it is important to note that the disparity in gender distribution in Experiment 1 may potentially influence the results. It is recommended to consider this factor and ensure a balanced gender representation in future studies to minimize any potential gender-related confounds.

An important implication of the present study is that the effect of intensity modulation was observed consistently in both the EEG and fNIRS signals, supporting the concept of neurovascular coupling. This coupling suggests that the electrical activity in the brain leads to an increase in arterial blood flow to meet the increased metabolic demands of postsynaptic activation and action potentials. This results in an oversupply of CBF, leading to an increase in HbO concentrations and a decrease in HbR, which can be measured using fNIRS (Attwell et al. 2010; Pinti et al. 2020). Furthermore, the hemodynamic response graphs in the present study show a delay of approximately 4 to 8 s after the onset of the stimulus, which is consistent with previous literature findings (van de Rijt et al. 2018). These results highlight the utility and complementary nature of EEG and fNIRS techniques, with fNIRS providing superior spatial resolution and EEG offering better temporal resolution.

Finally, taking into account the results of the present study, it could be suggested that fNIRS is a valuable tool for investigating activation and modulation responses to auditory stimuli. Although the statistical approach for fNIRS may be less robust compared to EEG, it offers a higher spatial resolution. Future studies should aim to address the limitations of fNIRS in auditory stimulation paradigms by implementing more effective signal filtering techniques to reduce physiological artifacts and using a wider range of decibel differences in the intensity of auditory stimuli. By addressing these considerations, researchers can enhance the potential of fNIRS for investigating auditory processing in greater detail.

## Conclusion

The present study provides evidence for the presence of intensity-dependent responses in the brain, as detected by both EEG and fNIRS, supporting the idea of neurovascular coupling in an auditory paradigm. These results suggest that the simultaneous recording of both EEG and fNIRS would benefit from the good time resolution of EEG, and the space resolution of fNIRS, given that the neurovascular coupling suggests that both signals are functionally related. Overall, this study contributes to our understanding of how electrical and hemodynamic signals work together in auditory paradigms, emphasizing the potential benefits of integrating EEG and fNIRS in future research.

### Supplementary Information

Below is the link to the electronic supplementary material.

Supplementary file1 (DOCX 1389 KB)

## Data Availability

The dataset generated and analyzed in the present study is available under reasonable request to the corresponding author (lmunnoz@us.es).
