# Supplementary material for: Neurovascular coupling during auditory stimulation: event-related potentials and fNIRS hemodynamic
Source: Brain Struct Funct. 2023 Sep 2;228(8):1943–61. doi: 10.1007/s00429-023-02698-9 (PMC10517045; doi:10.1007/s00429-023-02698-9)
Supplement: Supplementary file 1 — Supplementary file1 (DOCX 1389 KB) [file 429_2023_2698_MOESM1_ESM.docx]

**Supplementary Figures**


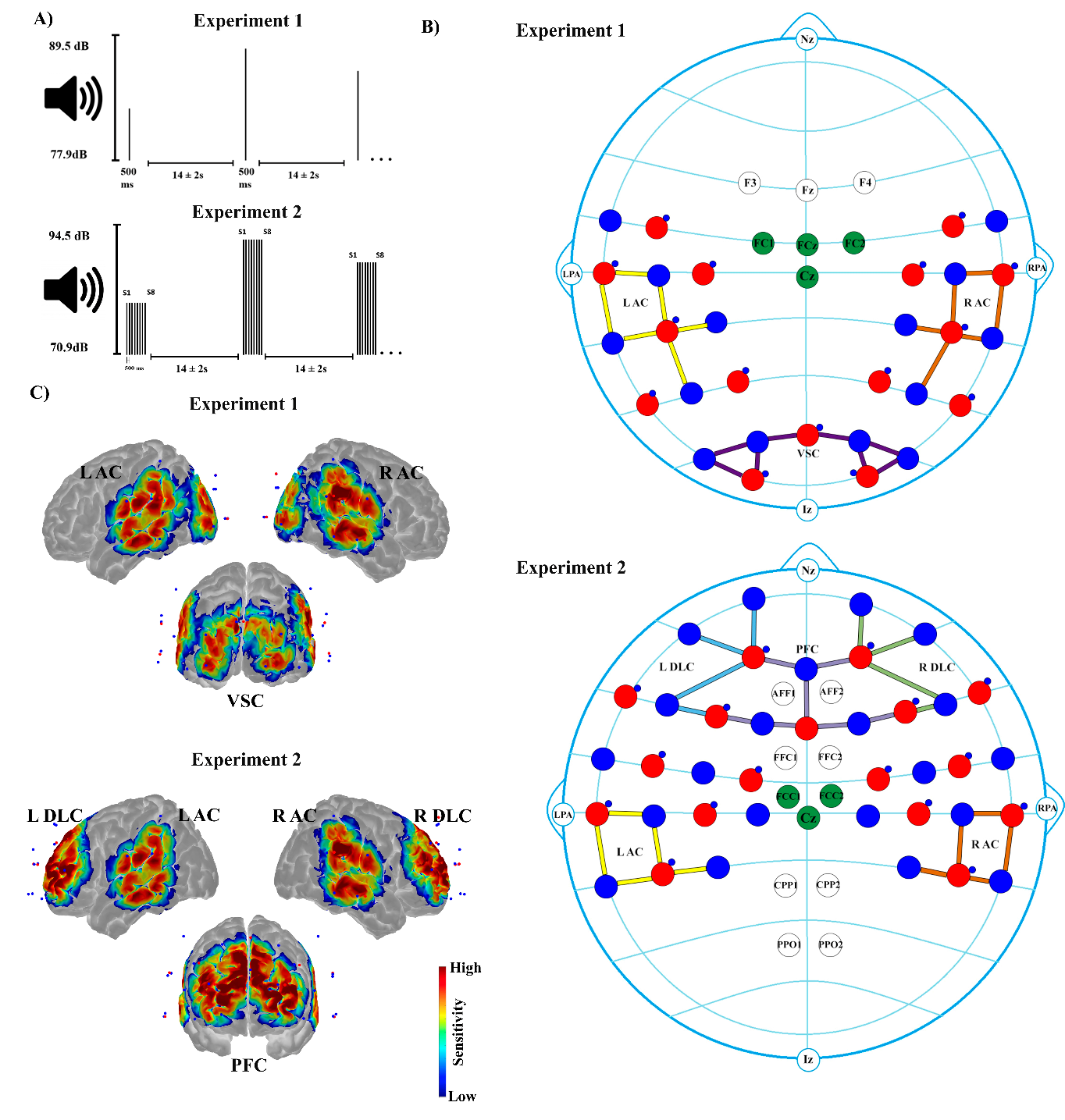


**Supplementary Fig. 1** A) Experimental protocol for the stimuli presentation. B) fNIRS and EEG montage of the experiments, showing the channels analyzed, selected with the software FOLD. The red circles correspond to the location of light sources, the blue circles are the light detectors, the blue small circles are the light detectors conforming short channels, and the green circles are the EEG central electrodes selected for the analysis. B) Sensitivity profile for the fNIRS channels analyzed extracted from Atlasviewer software. (L AC: Left auditory cortex, R AC: Right auditory cortex, VSC: Visual cortex)


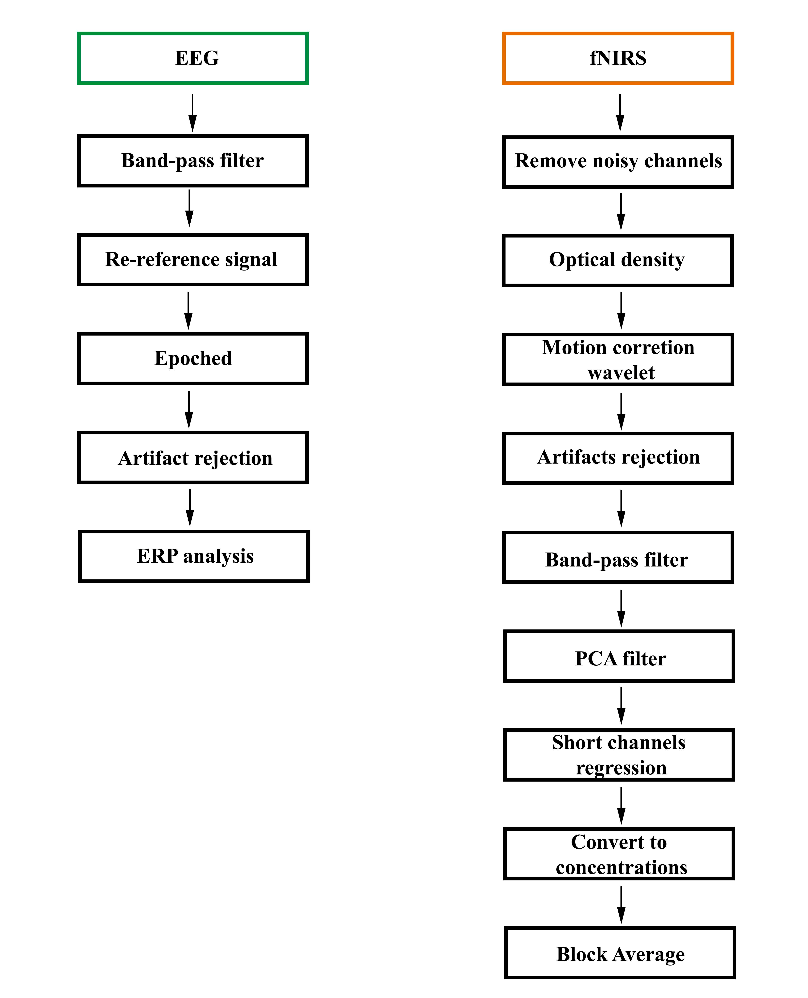


**Supplementary Figure 2.** Pipeline of EEG and fNIRS signal processing.


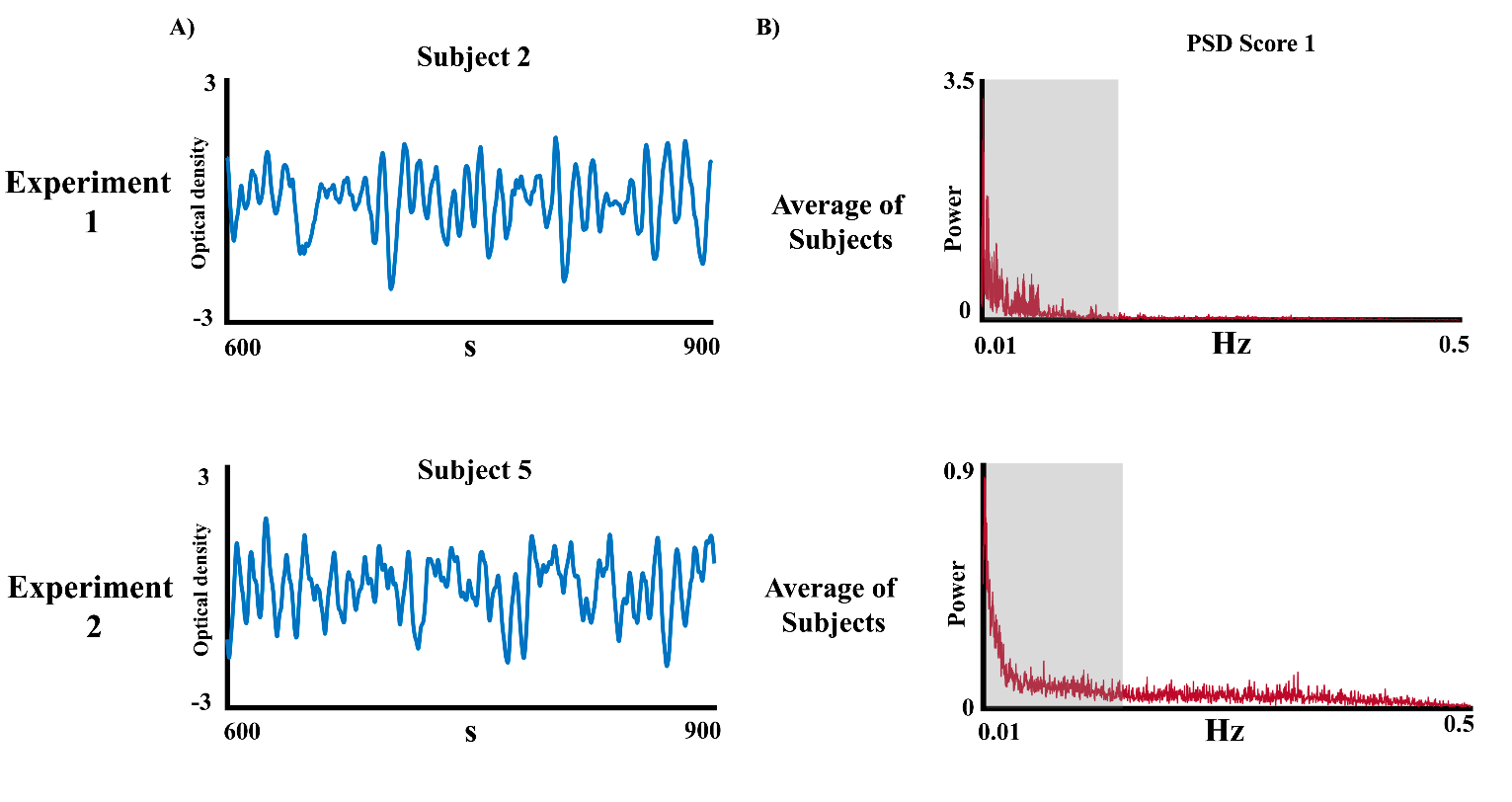


**Supplementary Fig. 3** Example of component extraction with PCA A) Plot of the scores of the first component extracted from the fNIRS signal by Principal Component Analysis (PCA) for two subjects of experiments 1 and 2. B) Average spectral power of the subjects, for the first component extracted from the PCA of the fNIRS signal through the HOMER2 function *enPCAFilter.* The grey area represent the LF power (0.01-0.15Hz).


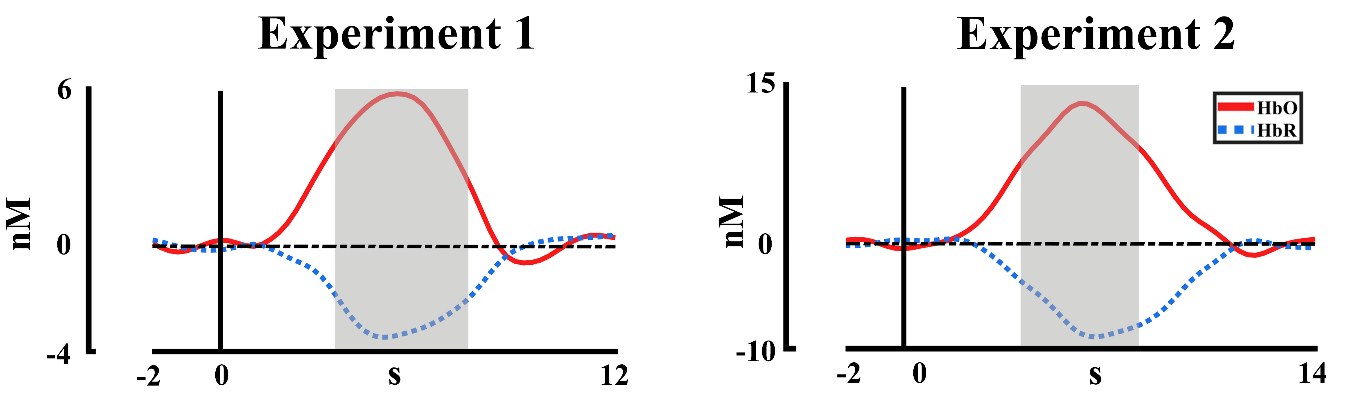


**Supplementary Fig 4.** Mean of the ROIs and intensity for the time windows selection process in both experiments. The time window selected for statistical analysis is marked in grey
